# From Early Adversity to Neurodegeneration: Stress Biomarkers as Predictive Signals for Lifespan Brain Health

**DOI:** 10.3390/ijms262211013

**Published:** 2025-11-14

**Authors:** Kenny Lemus-Roldan, Fabiola Castorena Torres, Daniela León Rojas, Julieta Rodríguez-de-Ita

**Affiliations:** 1 Escuela de Medicina y Ciencias de la Salud, Tecnologico de Monterrey, Ave. Morones Prieto 3000, Monterrey 64710, N.L., Mexico; 2 Escuela de Medicina y Ciencias de la Salud, Tecnologico de Monterrey, Hospital San José, TecSalud, Monterrey 64710, N.L., Mexico

**Keywords:** adverse childhood experiences, neurodegeneration, benevolent childhood experiences, health

## Abstract

Neurodegenerative diseases (NDs) represent a growing public health challenge worldwide. While their clinical manifestations typically emerge late in life, increasing evidence suggests that biological vulnerability may originate much earlier in life. Early childhood adversity, expressed through mechanisms of toxic stress and allostatic load, has been associated with chronic activation of the hypothalamic–pituitary–adrenal axis, mitochondrial dysfunction, oxidative stress, and persistent inflammation—molecular pathways that overlap with those implicated in neurodegeneration. This narrative review highlights recent advances linking early adversity with long-term brain health. It discusses stress-related biomarkers, such as hair cortisol, inflammatory cytokines, and epigenetic modifications, as potential early indicators of neurodegenerative risk. Remarkably, protective and benevolent childhood experiences may mitigate these biological trajectories, underscoring the role of resilience in shaping neurobiological outcomes. We argue that integrating pediatric cohorts, particularly in underrepresented regions such as Latin America, with longitudinal biomarker approaches and omics technologies offers a unique opportunity to identify early predictors and preventive strategies. Understanding neurodegeneration as a lifespan process opens new avenues for early intervention and public health policy.

## 1. Introduction

Nowadays, neurodegenerative diseases (NDs) present a significant burden and an increasing challenge for public health worldwide. As life expectancy steadily rises, the prevalence of conditions such as Alzheimer’s disease (AD) is expected to increase dramatically over the coming decades [1]. AD alone already affects millions of people globally and is the most common cause of dementia, with aging being one of the primary risk factors [2]. Currently, more than 55 million people worldwide have a dementia diagnosis, with 66% of them residing in low- and middle-income countries [3]. In the United States, approximately 7.2 million people aged 65 and older live with AD, accounting for 11% of the population in that age group. AD is the sixth-leading cause of death globally and the most prevalent ND, followed by Parkinson’s disease (PD) [3,4]. Analyses from the Global Burden of Disease, Injuries, and Risk Factors Study estimate that 6.1 million people worldwide have PD, with a rate of 1081 per 10,000 in Latin America and the Caribbean, and projections suggest that 25.2 million people will be living with PD by 2050 [5,6].

Regarding Multiple Sclerosis (MS), 1.89 million people live with it globally, with over 23.9 cases per 100,000 population. North America and Western Europe report the highest prevalences [7,8]. Meanwhile, amyotrophic lateral sclerosis (ALS) and Huntington’s disease (HD) are significantly less common, with 33,000 cases recorded in 2022 in the U.S. and 2.7 cases per 100,000 worldwide, respectively [9,10].

NDs are more common in the older population due to brain aging, which involves neurostructural and neurofunctional changes, as well as neuroinflammation. These processes have been associated with gradual cognitive decline and can occur even before clinical symptoms appear [1]. In this context, current research and prevention strategies have increasingly adopted a life-course perspective, recognizing that the origins of neurodegenerative disease risk start long before clinical onset. Evidence suggests that optimal brain development during gestation and early infancy supports cognitive reserve. Conversely, early exposures to adversity—such as malnutrition, toxic stress, or psychosocial deprivation—may biologically embed vulnerability decades before symptoms appear [11,12,13]. Within this framework, early childhood adversity has gained attention as an additional, potentially modifiable risk factor that may affect the brain’s vulnerability to neurodegeneration [14].

Several studies have linked adverse childhood experiences (ACEs), which include experiences of abuse, neglect, and household dysfunction within the first 18 years of life, to long-term health risks [15]. These experiences trigger a cascade of multisystemic changes that aim to maintain homeostasis but accumulate into physiological strains, also known as allostatic load [16].

The allostatic load resulting from childhood adversity triggers neuroendocrine and neuroinflammatory pathways, causes oxidative stress, and can even induce epigenetic modifications that can alter gene expression profiles. These biological effects are strongly linked to chronic physical and mental health diagnoses such as obesity, cardiovascular risk, depression, irritable bowel syndrome, and, more recently, cognitive decline and neurodegenerative diseases like AD, PD, and MS [15,16].

Neuroinflammation has been associated with cognitive impairment by affecting neurogenesis and synaptic plasticity, two essential processes for learning and memory [17]. Structural imaging studies further support these associations, showing that individuals exposed to ACEs exhibit widespread reductions in gray and white matter that persist into middle age. These findings highlight the role of ACEs in neurodegeneration and stress the importance of viewing neurodegeneration from a lifespan perspective rather than solely focusing on age-related changes [18].

The lasting impact of ACEs on the brain may overlap with or even accelerate natural brain aging. This highlights ACEs as crucial preventable risk factors for NDs [18]. Importantly, not all childhood experiences increase vulnerability; evidence shows that positive or benevolent childhood experiences (BCEs) are strongly associated with resilience and can reduce the impact of ACEs on health [19]. BCEs help foster healthier pathways into adulthood and old age. This makes them an even more vital focus for public health efforts, not only to support normal childhood development but also to encourage healthier adulthood and aging [20,21].

Beyond psychosocial associations, growing evidence shows that childhood experiences can become biologically embedded through measurable changes in stress-related biomarkers. These include markers associated with allostatic load and its underlying systems, such as hormones like cortisol (from the hypothalamic–pituitary–adrenal or HPA axis), inflammatory cytokines, oxidative stress markers, and proteins linked to neurodegeneration, among others [13,22]. To date, most research on stress-related biomarkers and neurodegeneration has focused on AD, PD, and even MS, because of their higher prevalence and well-defined biomarkers; however, the underlying mechanisms are probably shared across multiple neurodegenerative diseases. Tracking these biomarkers over the lifespan could serve as a translational bridge connecting early environmental exposures with long-term brain health and neurodegenerative disease risk. In this context, biomarkers provide an objective interface to identify biological mechanisms that contribute to vulnerability and resilience [13,23].

While studies like Nahar et al. elucidate psychosocial pathways linking ACEs and BCEs with mental well-being, and some other reviews address the relationship between ACEs and AD or subjective cognitive decline [14,21,24], to our knowledge, this is the first integrative review to explore the biological embedding mechanisms underlying these associations. It emphasizes lifespan stress-related biomarkers that may serve as early predictors of NDs. Although most biomarker data come from the most common NDs, the mechanisms discussed here represent shared biological processes underlying multiple neurodegenerative conditions. Understanding neurodegeneration as a lifespan process opens new possibilities for prevention, early intervention, and public health policies that recognize childhood as a critical period for lifelong health.

This review aims to highlight current evidence linking early adversity to neurodegeneration and long-term brain health. It discusses the main pathways through which ACEs may accelerate neurodegeneration, including stress-related, neuroendocrine, inflammatory, and epigenetic mechanisms. The review also summarizes evidence on stress-related biomarkers that could serve as early indicators of neurodegenerative risk. Additionally, we examine potential pathways by which BCEs promote resilience and protect against the biological effects of ACEs and propose directions for future research.

## 2. Methods

This narrative review was developed following the SANRA (Scale for the Assessment of Narrative Review Articles) guidelines and structured according to the PICO framework, refined through the Elicit artificial intelligence tool (elicit.org) to identify relevant studies addressing the biological mechanisms and stress-related biomarkers linking ACEs to neurodegeneration, while also integrating data on protective experiences and BCEs that may confer resilience through similar pathways.

Comprehensive searches were conducted in Elicit, PubMed/MEDLINE, Scopus, and Web of Science, with no date restrictions and updated through October 2025. The search combined MeSH terms and the following keywords: “Adverse childhood experiences” OR “Early life stress” OR “Allostatic load” OR “Positive childhood experiences” OR “Benevolent childhood experiences” OR “Psychosocial experiences” OR “resilience” AND “Hypothalamic-pituitary-adrenal axis” OR “Neuroinflammation” OR “cytokines” OR “mitochondrial dysfunction” OR “oxidative stress” OR “proteostasis” OR “protein aggregation” OR “genomics” OR “epigenomics” OR “transcriptomics” OR “proteomics” OR “metabolomics” OR “multiomics” OR “biomarkers” AND “neurodegeneration” OR “neurodegenerative diseases” OR “Alzheimer” OR “Parkinson” OR “multiple sclerosis” OR “amyotrophic lateral sclerosis” OR “Huntington disease” OR “brain aging.” All references were imported into Elicit’s library, duplicates were removed, and titles and abstracts were manually screened for relevance. Inclusion criteria included human observational, longitudinal, or interventional studies, as well as systematic or narrative reviews and meta-analyses reporting on molecular or omic outcomes and biomarkers associated with ACEs or BCEs, examining biological mechanisms related to neurodegeneration or aging. Studies had to be published in English or Spanish and be available in a full-text format. Exclusion criteria included animal or in vitro studies without translational validation, editorials, or theoretical commentaries. Due to the heterogeneity of study designs and outcomes, data were synthesized narratively and grouped along the following axes: 1. ACEs and biological embedding, 2. Shared molecular mechanisms between ACEs and neurodegeneration, 3. Stress-related biomarkers, 4. BCEs and biological mechanisms of protection. The results were integrated to demonstrate how early adversity biologically embeds risk for neurodegeneration, while BCEs and resilience mechanisms may buffer these effects through neuroendocrine, inflammatory, epigenetic, and bioenergetic modulation.

## 3. Early Childhood Adversity and Biological Embedding

Early-life adversity—from the perinatal period through the first few years of childhood—shapes developmental paths far beyond the behavioral domain. Stressors faced during sensitive periods leave lasting biological “footprints,” a process called biological embedding, where toxic stress and ongoing allostatic load adjust neuroendocrine, immune, and metabolic systems throughout life. Even in non-clinical pediatric groups, combined measures of allostatic load have been linked to worse physical and cognitive outcomes, showing that vulnerability appears before clear signs of illness [25].

Hypothalamic–pituitary–adrenal (HPA) axis dysregulation is a key pathway in this process. Meta-analytic evidence links adversity to alterations in cortisol’s daily rhythm, blunted or disorganized reactions to acute stress, and increased markers of chronic stress like hair cortisol, with patterns differing by age, type of adversity (threat versus deprivation), and timing of exposure [15]. These results support the idea of a lasting “stress recalibration” of the HPA set point, which extends beyond childhood. Hair cortisol studies also suggest a phenotype characterized by heightened psychological reactivity combined with a blunted endocrine response, a combination associated with increased risk for psychopathology and later somatic dysregulation [26].

Along with endocrine changes, immune-inflammatory pathways are also activated. Children exposed to early adversity show low-grade inflammation and higher circulating biomarkers—such as C-reactive protein (CRP), interleukin-6 (IL-6), and tumor necrosis factor-alpha (TNF-α)—even outside clinical settings [27]. This immune activation constitutes a plausible link to increased cerebrovascular vulnerability, synaptic remodeling, and accelerated neurodegenerative processes.

A third pathway involves oxidative stress and redox imbalance, which interact with HPA and immune responses. Perinatal psychosocial stress has been associated with elevated levels of F2-isoprostanes, markers of lipid peroxidation, and altered cognitive profiles in infants and preschoolers [28]. Although effect sizes are modest and occasionally inconsistent, the biological signal is evident. A 2024 review emphasizes the translational importance of oxidative markers, including 8-OHdG and F2-isoprostanes, in pediatric populations, highlighting their role in cumulative cellular damage [28,29].

At the molecular level, epigenetic regulation functions as a long-term “recording system” of experience. Changes in DNA methylation that affect genes involved in synaptic plasticity, neuroinflammation, and energy metabolism have been linked to adversity and can last for decades, with early evidence of intergenerational effects [30]. A 2024 review also highlights sex-specific and lasting changes involving HPA–immune interactions and molecules crucial for fronto-limbic development, underscoring sensitive periods for neural circuit formation [31].

These biological pathways converge on neurocognitive function. A 2024 cohort showed that adversity patterns—whether diffuse or domain-specific—relate to differences in executive processes, supporting the dimensional model of threat and deprivation [32]. Functional neuroimaging supports these results: adolescents with higher ACEs scores demonstrate altered blood oxygen level-dependent activity in regions involved in executive control and emotion regulation networks, particularly within the prefrontal cortex and hippocampus, areas known for their stress sensitivity [33]. Consequently, disruptions in self-regulatory systems emerge, compromising working memory and inhibitory control and raising the likelihood of maladaptive behaviors such as emotional eating [34].

Beyond the brain, early adversity affects metabolic programming. Sympathetic overactivity, insulin resistance, endothelial dysfunction, and chronic inflammation form converging pathways linking ACEs to cardiometabolic risk [35,36]. Pediatric obesity and early clustering of metabolic risk factors worsen vascular stress and—when combined with embedded stress biology—increase vulnerability to long-term neurological issues [37].

Finally, growing evidence indicates that adversity might prime the brain for neurodegeneration, creating a latent biological vulnerability. Signals from HPA dysregulation, immune activation, oxidative stress, and epigenetic remodeling may reduce resilience and make neural systems more prone to synaptic decline and cognitive impairment decades later in life [18]. Using standardized biomarkers such as hair cortisol, inflammatory panels (IL-6, CRP, TNF-α), and oxidative markers (8-OHdG, F2-isoprostanes) could strengthen translational bridges between ACEs screening and preventive monitoring of neurocognitive and metabolic risks across development [25,27,29].

## 4. Adversity Shared Molecular Mechanisms with Neurodegeneration

Neurodegenerative diseases present major health challenges worldwide, with their prevalence increasing rapidly in aging populations. Although these conditions usually emerge clinically later in life, increasing evidence indicates that their molecular roots may form much earlier, even during childhood.

**Figure 1:**
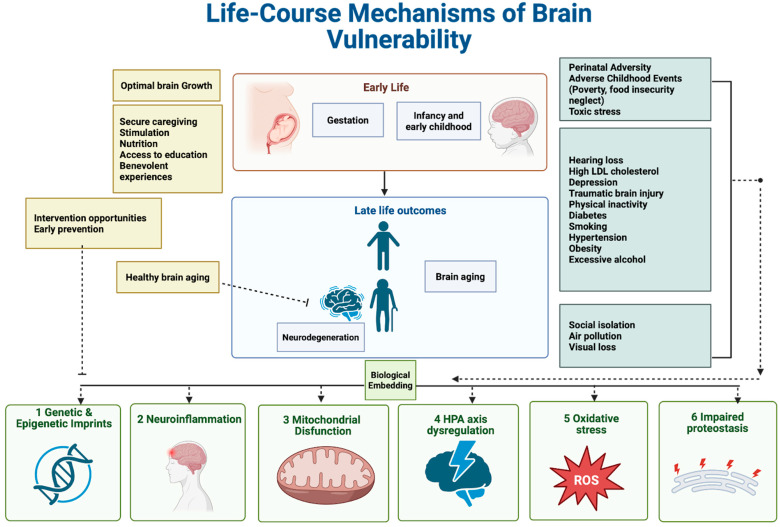
Life-course biological mechanisms linking early adversity to neurodegeneration.

ACEs and toxic stress have significant biological effects that influence multiple physiological systems. These effects overlap with key mechanisms of neurodegeneration, such as neuroinflammation, mitochondrial dysfunction, HPA axis dysregulation, oxidative stress, and disrupted proteostasis (Figure 1). Recognizing these shared molecular pathways shifts ND’s perspective from conditions solely associated with aging to those initiated by processes that begin decades earlier, highlighting the potential for prevention efforts to begin in childhood [1,18].

This framework illustrates how protective conditions—such as sensitive caregiving, proper nutrition, cognitive stimulation, access to education, and benevolent experiences—support optimal brain development and cognitive reserve. Conversely, adverse childhood experiences (ACEs)—including poverty, neglect, food insecurity, and toxic stress—cause lasting biological embedding beginning early in life. Six interconnected mechanisms come into play: (1) adversity triggers epigenetic imprint in stress regulation genes, like methylation of GR/NR3C1 and FKBP5, along with altered CRH–ACTH–cortisol signaling and increased hippocampal vulnerability; (2) neuroinflammation and glial priming, characterized by chronic cytokine signaling (IL-1β, IL-6, TNF-α), microglial and astrocytes with elevated GFAP and TSPO expression, blood–brain barrier dysfunction, and synaptic disruption; (3) mitochondrial dysfunction and impaired organelle quality control, including deficits in oxidative phosphorylation, buildup of reactive oxygen species (ROS), damage to mitochondrial DNA, changed mitochondrial dynamics (via DRP1, MFN1/2, OPA1), and faulty mitophagy involving PINK1/PRKN; (4) dysregulation of the hypothalamic–pituitary–adrenal (HPA) axis dysregulation, with impaired feedback presenting as either chronic hypercortisolemia or decreased stress responsiveness, along with peripheral biomarkers such as elevated HCC); (5) oxidative stress and cellular aging, driven by ROS imbalance, DNA damage markers like 8-OHdG, lipid peroxidation, telomere shortening, and inadequate NRF2–antioxidant responses; and (6) disrupted proteostasis and protein aggregation, with maladaptive unfolded protein responses in the endoplasmic reticulum, reduced autophagy–lysosomal and ubiquitin–proteasome system activity, and accumulation of proteins such as Aβ, phosphorylated tau, and α-synuclein. Early adversity heightens vulnerability to mid- and late-life risk factors—including hypertension, diabetes, smoking, depression, social isolation, pollution, and hearing loss—thereby increasing the risk of neurodegeneration. Protective factors like education, secure attachment, and ongoing physical and cognitive activities can buffer these effects and promote healthy brain aging.

### 4.1. Inflammation and Immune Dysregulation

One of the most consistent pathways linking ACEs to neurodegeneration is immune activation. Children and adolescents who face early adversity show elevated inflammatory markers such as IL-6, TNF-α, and CRP. These markers often persist into adulthood as signs of chronic, low-grade inflammation and are also central to neurodegenerative diseases like MS [38,39,40,41].

In neurodegenerative diseases, activated microglia secrete pro-inflammatory cytokines, contributing to synaptic pruning, neuronal death, and circuit disruption [1]. Chronic systemic inflammation caused by ACEs may condition microglial populations toward a persistently pro-inflammatory state, thereby lowering the activation threshold for future pathological stimuli and facilitating the progression of neurodegenerative processes. In PD, microglial dynamics reveal a self-perpetuating cycle involving α-synuclein accumulation, mitochondrial damage, and chronic inflammation, which accelerates the degeneration of vulnerable neurons, a pattern conceptually compatible with adversity-induced inflammation [38].

Neuroimaging evidence further supports this connection. Positron Emission Tomography (PET) tracers targeting translocator protein, a mitochondrial protein upregulated in activated microglia, have allowed visualization of neuroinflammation in AD and PD, although limitations in specificity and genetic polymorphisms complicate interpretation. Alternative targets are emerging to enhance precision in tracking inflammatory processes [39]. Collectively, these findings suggest a “two-hit model”: early adversity establishes a pro-inflammatory baseline. Simultaneously, aging-related insults or genetic susceptibility serve as the second hit, leading to overt neurodegenerative pathology [42].

### 4.2. Mitochondrial Dysfunction and Allostatic Load

Mitochondria are highly sensitive to psychosocial stress and play a crucial role in linking adversity with long-term brain health. Proteomic studies in adults with ACEs show significant changes in mitochondrial pathways, including a 1.5–2.2-fold upregulation of oxidative phosphorylation proteins and higher expression of immune-related mitochondrial proteins, indicating increased metabolic demand and mitochondrial allostatic load [42,43].

Prolonged exposure to glucocorticoids (GCs), a hallmark of chronic HPA-axis dysregulation, disrupts mitochondrial homeostasis by reducing complex I activity and ATP production by approximately 30–40%, while also increasing mitochondrial ROS levels and mPTP (mitochondrial permeability transition pore) opening by nearly 80%. This mitochondrial damage coincides with a 2–3-fold increase in phosphorylated Tau, establishing a mechanistic link between stress signaling and AD pathology [44,45]. In frontotemporal dementia (FTD), hyperphosphorylated tau driven by CDK5 and GSK3β causes microtubule destabilization and neurofibrillary pathology [46,47]. Although direct evidence connecting GC–GR activation to tau pathology in FTD is emerging, glucocorticoid signaling is known to promote GSK3β/CDK5-dependent tau phosphorylation in tauopathic models [48], indicating a stress-sensitive mechanism relevant for FTD progression. In dementia with Lewy bodies (DLB), Tau hyperphosphorylation destabilizes microtubules, impairs axonal transport, and promotes neurofibrillary tangle formation. This process spreads across vulnerable networks and synergizes with α-synuclein to worsen synaptic dysfunction and cognitive decline [49].

In PD, α-synuclein directly interferes with oxidative phosphorylation, leading to bioenergetic deficits [50]. In DLB, α-synuclein aggregation is the primary pathological feature, forming Lewy bodies and Lewy neurites that spread in a prion-like fashion through cortical and subcortical regions. Misfolded α-synuclein damages mitochondrial and proteostatic pathways, causing synaptic dysfunction and the distinctive cognitive and motor symptoms of DLB. Interactions with Aβ and Tau further enhance aggregation and toxicity [49]. Failure to eliminate damaged mitochondria via the stress-sensitive PINK1/Parkin mitophagy pathway contributes to neurodegeneration, with basal mitophagy reduced by 30–50% in the hippocampus of Alzheimer’s patients, along with noticeable decreases in PINK1 and Parkin expression. Stress-related activation of the HPA axis has been shown to suppress mitophagy-related gene expression, suggesting a mechanistic bridge between ACEs and later mitochondrial vulnerability [51,52].

Preclinical models support these findings: maternal separation paradigms cause mitochondrial ultrastructural alterations, including cristae swelling, matrix rarefaction, and impaired oxidative phosphorylation in rodent hippocampal neurons. Human evidence mirrors this vulnerability, as early-life adversity predicts accelerated neuroinflammation and neurodegeneration, demonstrated by age-dependent increases in serum GFAP (F = 3.22, *p* = 0.012) and NfL (F = 11.24, *p* < 0.001), along with a progressive reduction in total and gray matter volume (total gray matter volume, F = 6.84, *p* = 0.001; total brain volume, F = 5.10, *p* = 0.006) and an enlargement of the third ventricle (F = 4.98, *p* = 0.001). These multimodal changes mirror early structural and molecular features seen in AD [1]. Finally, although not currently linked to ACEs, in ALS, mutant SOD1 (Superoxide dismutase 1) interacts directly with the mitochondrial voltage-dependent anion channel 1, disrupting oxidative phosphorylation and increasing ROS buildup, while in HD, mutant huntingtin impairs mitochondrial fission-fusion dynamics and lowers ATP production, worsening neuronal vulnerability to oxidative stress [53]. More evidence is needed to confirm the connection between these pathologies and ACEs. However, evidence suggests that ACE-related mitochondrial dysfunction may accelerate biological aging trajectories, effectively causing premature mitochondrial aging and reducing resilience to subsequent neurodegenerative insults.

### 4.3. HPA Axis Dysregulation and Epigenetic Imprints

The HPA axis functions as the key interface between environmental stress and neuroendocrine activity. Early adversity has been associated with ongoing dysregulation of the HPA axis, manifesting as either chronic hypercortisolemia or attenuated stress reactivity, depending on the type and developmental timing of exposure [42].

A dysregulated HPA axis has been observed in MS, with over 50% of patients exhibiting high cortisol levels and immune cells showing increased resistance to glucocorticoids [41].

At the molecular level, early adversity causes epigenetic alterations in stress regulation genes, especially NR3C1 and FKBP5, which are among the most studied targets in human trauma research, showing consistent NR3C1 hypermethylation and FKBP5 hypomethylation [30]. Epigenetic silencing of glucocorticoid receptors decreases feedback inhibition, leading to prolonged cortisol exposure. Chronic GC signaling is neurotoxic, impairing hippocampal plasticity, reducing dendritic arborization, and increasing amyloid-β deposition and tau phosphorylation in AD [43]. FKBP5 dysregulation also modulates GC receptor sensitivity and has been implicated in both stress-related psychiatric disorders and neurodegenerative diseases [54].

In DLB, amyloid-β (Aβ) accumulation is amplified by stress-related HPA-axis activation, in which glucocorticoid receptor signaling increases amyloidogenic APP processing by upregulating BACE1 and enhancing PSEN1 activity. This promotes excess Aβ production and deposition, which is clinically relevant in DLB, where mixed Aβ/α-syn pathology accelerates synaptic deterioration and cognitive decline. Impaired glymphatic and vascular clearance further contributes to plaque burden and synaptic vulnerability [49].

The molecular imprints of early adversity cause long-lasting changes in stress-response pathways, embedding vulnerability into neuroendocrine and immune regulation, and ultimately triggering neurodegenerative cascades throughout the lifespan.

### 4.4. Oxidative Stress and Cellular Senescence

Oxidative stress acts as a key convergence pathway. Individuals exposed to ACEs exhibit higher levels of oxidative damage biomarkers, including 8-OHdG and malondialdehyde (MDA), a lipid peroxidation byproduct, along with reduced antioxidant defenses [29]. These changes speed up telomere shortening and mitochondrial DNA (mtDNA) depletion, both of which are known as strong indicators of biological aging [43].

In AD and PD, oxidative stress worsens pathology: amyloid-β deposits, tau aggregates, and α-synuclein accumulation increase ROS production, creating a vicious cycle of cellular damage and impaired repair [43,45]. Also in MS, oxidative stress is a key factor associated with demyelination through mitochondrial ROS generation, lipid peroxidation of myelin membranes, and release of inflammatory cytokines [55]. The shared oxidative phenotype indicates that toxic stress accelerates cellular aging processes, thereby decreasing resilience to neurodegenerative insults encountered later in life [39,43].

### 4.5. Protein Aggregation and Impaired Proteostasis

Chronic stress also disrupts proteostasis. Stress-induced mitochondrial dysfunction and inflammation impair autophagy and the ubiquitin-proteasome system, promoting the accumulation and spread of misfolded proteins.

In AD, glucocorticoids enhance the secretion and transneuronal spread of phosphorylated tau through a GSK3β-dependent non-canonical pathway, providing a direct mechanism by which stress accelerates the pathological dissemination of tau in AD [54]. In PD, microglia internalize, process, and re-release pathological α-synuclein via phagocytic and endosomal–lysosomal pathways, facilitating its cell-to-cell trafficking. This process amplifies neuroinflammation—driven by cytokine signaling, ROS production, and NF-κB activation (Nuclear Factor kappa-light-chain-enhancer of activated B cells)—thus strengthening glia–neuron feedback loops that promote the aggregation, propagation, and pathological dissemination of misfolded proteins across vulnerable neural networks [56].

Dysregulation of proteostasis is also central to ALS and HD, where protein aggregation (TDP-43, SOD1, and mutant huntingtin) spreads through prion-like mechanisms, leading to progressive neuronal loss and glial activation. Chronic stress and HPA-axis hyperactivation can worsen these failures by depleting heat-shock proteins and impairing autophagic clearance [53]. Although these mechanisms in the latter pathologies have not been directly linked to ACEs, they are relevant because they illustrate direct mechanisms by which stress accelerates protein aggregation and pathological spread, which are hallmarks of neurodegeneration. This suggests potential for future translational research.

### 4.6. Brain Structure, Biomarker Evidence of Adversity and Neurodegeneration Shared Mechanisms

Neuroimaging provides macroscopic evidence of some of the previously discussed mechanisms. Children exposed to ACEs show reduced hippocampal and prefrontal volumes, altered white matter integrity, and increased amygdala reactivity [1,38]. These regions overlap with those most affected in AD and PD.

In older adults, ACE exposure is associated with poorer cognitive performance and less favorable neuroimaging profiles, as well as plasma biomarkers related to dementia risk, including serum neurofilament light chain (NfL), glial markers, and cytokine panels [1,52,57]. NfL, a reliable biomarker of axonal damage, has been connected to both psychiatric and cardiovascular risks, making it a bridge biomarker that connects stress biology with neurodegenerative vulnerability [57].

Taken together, the evidence shows that ACEs and toxic stress converge on a biological triad related to neurodegeneration by (1) HPA dysregulation, sustained glial inflammation and cytokine signaling, (2) mitochondrial dysfunction and impaired mitophagy, and (3) aggregation and propagation of pathological proteins (tau, α-synuclein). These processes, detectable through inflammatory imaging, circulating biomarkers, and omics data, illustrate how early life adversity lays the foundation for neurodegeneration. Recognizing this convergence redefines dementia as the result of life-course processes, not just a disease of old age, and highlights the need for preventive interventions targeting these mechanisms and pathways decades before symptoms appear. A summary of the main molecular and cellular mechanisms discussed above, together with their associated biomarkers and disease relevance, is presented in Table 1.

## 5. Stress-Related Biomarkers as Early Predictors of ACEs Biological Effects

Identifying stress biomarkers allows early detection of the biological effects of ACEs and helps predict their impact on brain health throughout life. Recent research highlights various biological markers, from infancy to adulthood, that demonstrate how chronic stress becomes “embedded” in the body and relates to neurodegeneration.

### 5.1. Hair Cortisol Concentration (HCC)

In childhood, hair cortisol has served as a non-invasive marker of long-term exposure to endogenous glucocorticoids, indicating HPA axis activity over weeks to months. Recent longitudinal research shows that higher hair cortisol levels are linked to an increased burden of ACEs and alterations in executive functions and the immune system, even in children without clinical diagnoses [25,58,59,60,61].

A study conducted on preschool children found that higher hair-cortisol levels were associated with authoritarian and coercive parenting styles, even after considering sex, ethnicity, and stressful family events. This suggests that the early environment affects long-term cortisol exposure and that HCC could serve as an early marker of allostatic load [58]. Similarly, different types of adversity (deprivation versus threat) have been connected to HPA-axis activity, measured through hair cortisol. For instance, neglect has been linked to lower hair cortisol levels, indicating that the type of ACEs influences the biomarker signal [25].

### 5.2. Inflammation and DNA Methylation

Inflammatory and epigenetic mechanisms continue to emerge as plausible mediating pathways of the biological impact of adversity. Pro-inflammatory cytokines, such as IL-6, TNF-α, and high-sensitivity C-reactive protein indicate persistent, low-grade inflammation linked to early life stress, which predicts a higher risk of cardiometabolic disease, neurodegenerative conditions, and neuropsychiatric disorders later in life [37].

Overall, ACEs studies suggest that early exposure is associated with elevated inflammatory markers and epigenetic changes in genes like NR3C1 and FKBP5, although more recent population-based studies directly examining these biomarkers in childhood are still needed [27]. Additionally, DNA methylation of HPA-regulatory genes (for example, hypermethylation of NR3C1 and FKBP5) functions as an epigenetic mechanism of biological embedding, affecting the expression of glucocorticoid receptors and thus the stress response, which is associated with increased emotional reactivity and difficulties in self-regulation during childhood [30,62].

### 5.3. NfL and GFAP (Glial Fibrillary Acidic Protein)

In adulthood, markers of neurodegeneration reflect the trajectory of chronic stress. NfL, present in serum and plasma, is a sensitive indicator of axonal injury and shows early elevations in individuals with a high history of ACEs, even before clinical signs of cognitive decline [1,63,64]. Additionally, NfL levels predict whole-brain and thalamic atrophy in patients with MS. They are highly predictive of future progression, regardless of relapse activity, and serve as a supported biomarker for pediatric MS case follow-up [65]. In this context, GFAP, produced by reactive astrocytes, increases with chronic neuroinflammation and acts as a marker of astroglial injury, predicting the risk of dementia and MS progression, and is found in both cerebrospinal fluid and the bloodstream [65]. It appears promising to explore them in relation to early life adversity [1].

### 5.4. Tau and β-Amyloid

Classic Alzheimer’s disease biomarkers such as phosphorylated tau and β-amyloid (Aβ42/Aβ40) in plasma or cerebrospinal fluid are considered part of the same vulnerability axis. Evidence suggests that individuals with a history of early stress show tau and amyloid profiles consistent with a preclinical predisposition to dementia [66,67]. Therefore, highlighting their importance in exploring biomarkers for the shared mechanism of neurodegeneration and adversity.

### 5.5. Biomarker Continuum Across the Lifespan

The previous findings support examining a biomarker continuum that traces the “biological history” of adversity exposure, from HPA-axis hyperactivity and systemic inflammation in childhood, through epigenetic programming, to markers of neurodegeneration in adulthood. This comprehensive approach supports prevention and monitoring and opens the door to early interventions that can reduce the risk of cognitive decline and neurodegenerative diseases. Overall, stress-related biomarkers—such as hair cortisol, pro-inflammatory cytokines, and early-life DNA methylation—along with NfL, GFAP, tau, and amyloid in adulthood, serve as essential links between childhood adversity and brain health across the lifespan, representing vital tools for preventive and personalized medicine (Table 2).

## 6. Positive and Benevolent Childhood Experiences Neurobiological Pathways Through Resilience

Unlike ACEs, positive or benevolent childhood experiences (BCEs) offer support and encourage healthy development in children. These experiences include consistent home routines and emotional support from caregivers, encouragement from teachers, having beliefs that provide comfort, feeling comfortable with oneself, liking school, and having good neighbors, among others [68]. They have consistently been linked to resilience, a person’s ability to adapt to adversity, stay healthy, and recover after temporary setbacks [69]. Growing evidence shows that BCEs can buffer the harmful effects of ACEs, acting as protective factors against common mental health problems such as depression, post-traumatic stress disorder, and sleep issues. Importantly, BCEs may also contribute to the development of cognitive reserve and support long-term brain health [21,70]. In this way, BCEs could mitigate ACE-related changes that lead to neurodegeneration.

A small but growing body of research has begun to link BCEs with brain aging and biomarkers of neurodegeneration. Although the exact mechanisms by which BCEs impact NDs risk are not fully understood, one study on the association of positive childhood experiences (PCEs) with AD cognition and biomarkers suggests that a key pathway connecting PCEs to late-life cognitive outcomes is education, as PCEs increase educational attainment and promote better cognition, especially memory intercept. However, no associations were found between PCEs and amyloid PET burden or hippocampal volume [71]. In this context, greater years of schooling and higher educational quality are well-established contributors to cognitive resilience in older adulthood [71]. At the biomarker level, one cross-sectional study reported lower levels of tauopathy in tau PET scans among individuals who attended a private school in childhood compared to those who attended public school (*p* = 0.036) [72].

In a large Chinese cohort, individuals with deficits in childhood peer relationships had a higher risk of dementia (OR 1.21, 95% CI 1.10–1.34) compared to those who reported more positive experiences [73]. Additionally, in another study, lower levels of cerebrospinal fluid (CSF) p-tau 181 (*p* = 0.037) and plasma tau 217 (*p* = 0.029) were found among those who often felt loved, supported, protected, and close to their families and friends during adolescence [72].

A higher level of parental involvement leads to increased synaptic proliferation, a denser cortex, and greater intellectual flexibility, which help reinforce neuronal resistance to natural aging or potential brain damage, as seen with ACEs [74]. Supporting evidence indicates that early enrichment and positive maternal environments can directly influence neurodevelopmental outcomes at molecular and structural levels. Miguel et al. demonstrated that prenatal maternal exercise, postnatal environmental enrichment, and optimal nutrition (including supplementation with n-3 polyunsaturated fats) boost hippocampal brain-derived neurotrophic factor expression, increase dendritic spine density, and improve oxidative resilience, while also reducing stress-related HPA reactivity. These environmental experiences could counterbalance the detrimental effects of ACEs, fostering resilience against brain changes linked to the development of later psychopathology [75].

Given the hippocampus’s critical role in memory and its vulnerability to neurodegenerative processes, both ACEs and BCEs are believed to have lasting impacts on brain health [76]. As mentioned earlier, ACEs are associated with the constant activation of the HPA axis, increased cortisol release, and ongoing neuroinflammation. These biological changes can damage hippocampal health, accelerate neuronal aging, and decrease synaptic plasticity. Conversely, BCEs may help mitigate these risks by promoting emotional security, adaptive stress regulation, and strengthening the prefrontal and hippocampal circuits related to resilience [76].

Growing evidence shows that resilience is an active, biologically driven process involving neural mechanisms in the hippocampus, prefrontal cortex, and reward systems, supported by neuroplasticity, immune regulation, and the integrity of the blood–brain barrier [76,77]. Additionally, research indicates that the molecular and cellular regulation of ionic channels in dopaminergic neurons of the ventral tegmental area, the activity of transcription factors coordinating pro-resilience gene networks in the prefrontal cortex and nucleus accumbens, and neuroinflammatory modulation—including reduced infiltration of inflammatory markers, increased IL-10 levels, and better blood–brain barrier function—are essential in developing resilience and provide promising research targets [76,77]. These findings suggest that resilience is a dynamic process that can be nurtured and strengthened for preventive purposes, potentially helping to prevent neurodegeneration [78].

Translational evidence from Kentner et al. supports the idea of resilience priming, where enriched environments, high-quality care, and sensorimotor stimulation promote long-term neuroplasticity changes. These include increased hippocampal brain-derived neurotrophic factor expression, greater dendritic complexity, lower corticosterone reactivity, and anti-inflammatory cytokine profiles with elevated IL-10 levels and decreased TNF-α. Epigenetic regulation of glucocorticoid receptor expression (Cohen’s d = 0.8) further shows that resilience can be biologically embedded from early life, providing protection against later stress-related neurodegenerative pathways [79].

Although direct evidence linking BCEs to mitochondrial or bioenergetic pathways is still lacking, converging adult studies suggest that positive psychosocial experiences may exert comparable biological effects later in life. For example, Trumpff et al. found that individuals reporting more positive psychosocial experiences had significantly higher mitochondrial oxidative phosphorylation protein levels in the dorsolateral prefrontal cortex (B = 0.27, *p* = 0.004), especially within glial cells. These results offer evidence that psychosocial enrichment can support cellular energy homeostasis and glial resilience, indicating a possible molecular pathway through which positive early life environments could also provide neuroprotective benefits throughout the lifespan [80].

Considering the emerging evidence, promoting BCEs can be seen as an initial step in primary prevention of pathological aging and cognitive decline, emphasizing that strengthening protective biological mechanisms rather than merely reducing adversity offers a practical approach to NDs prevention [71,74,75].

However, important challenges still need to be tackled in future research, such as the need for replication in longitudinal, translational, and multimodal studies that combine molecular, circuit-level, and systemic findings with dependable behavioral paradigms. Prospective cohorts that assess both adversity and benevolent experiences during sensitive developmental periods are essential for understanding how these factors interact to affect brain health over a lifetime. Ultimately, addressing these research gaps will be crucial for creating preventive and therapeutic strategies that focus on resilience mechanisms, shifting the goal from simply reducing vulnerability to strengthening the brain’s biological adaptive capacity.

## 7. Future Directions

After reviewing the growing evidence linking childhood adversity to stress-related biomarkers and molecular pathways involved in neurodegeneration, it is important to consider the remaining gaps, future research directions, and regulatory and ethical implications.

Although progress in biomarker discovery has been significant, there remains a clear lack of robust clinical and longitudinal cohorts to validate the proposed links between ACEs and NDs, highlighting the need for prospective longitudinal studies. Turning initial findings into practical strategies requires a broader perspective that considers regional differences, long-term data, and prevention approaches.

### 7.1. Addressing Geographic and Methodological Gaps

A consistent limitation in the field is the overrepresentation of high-income countries in studies of adversity biomarkers. Most large-scale investigations on neurodegeneration and early-life stress come from Europe or North America, with limited inclusion of populations from low- and middle-income countries. This imbalance matters because the prevalence of ACEs, poverty, and psychosocial stressors is often higher in underrepresented regions like Latin America. Without region-specific data, our understanding of biological vulnerability risks remains incomplete and potentially biased. Recent publications from Latin America are starting to close this gap [81,82,83,84]. Studies have reported associations between childhood adversity and markers of inflammation, altered DNA methylation, and cognitive outcomes in children and adolescents. These findings confirm that the biological embedding of stress is not restricted to high-income settings but also happen in areas marked by inequality and limited healthcare access. Importantly, this emerging evidence shows that psychosocial factors like parental mental health, community violence, and neighborhood disadvantage interact with biological pathways, emphasizing the need to include social determinants in biomarker research [83].

### 7.2. Alignment with Global Frameworks

The previously emphasized need for regionally contextualized studies aligns with international frameworks that increasingly stress the importance of a lifespan approach. The American Academy of Neurology Brain Health Platform promotes shifting neurology toward prevention, highlighting the importance of promoting brain health throughout the entire life span [12]. The World Health Organization position paper on brain health expands this view by concentrating on cognitive, emotional, and social well-being from childhood to old age, with a clear focus on equity and inclusion [11]. The Lancet Standing Commission on Dementia Prevention estimates that up to 40% of dementia cases could be prevented or delayed by addressing modifiable risk factors, many of which begin early in life [85].

Together, these frameworks agree that effective prevention must begin well before clinical symptoms emerge. By placing Latin America’s emerging research within this global context, it becomes evident that building regional cohorts and integrating psychosocial and biological perspectives is both a scientific priority and a public health necessity.

### 7.3. Regulatory and Ethical Aspects

As research on this topic combines clinical records, genetic data, and digital health monitoring to identify early biomarkers of neurodegeneration, ethical and regulatory issues become critical. Using such data requires strict adherence to privacy and data protection laws like the Health Insurance Portability and Accountability Act, the General Data Protection Regulation, or local policies. Additionally, concerns about informed consent, data ownership, secondary use of biological samples, and protection against the identification of individuals from genetic or omic data remain central to ethical research practices. Moreover, as digital biomarkers and longitudinal health monitoring expand, developing frameworks for ethical data governance and fair access will be essential to ensure that intervention and prevention strategies derived from research are applied equitably and are adapted to different regions.

### 7.4. Policy Implications

Translating biomarker evidence into policy requires deliberate investment in early prevention. Strategies should aim to reduce childhood adversity, build resilience, and incorporate brain health into maternal and child health programs. Interventions must be multi-level, combining psychosocial support, nutritional initiatives, educational reforms, and community-based services. Including omics data collection within these frameworks would allow policymakers to monitor biological effects in real time and develop interventions that address both social and biological factors that impact health.

Such efforts would also boost global equity in science. Latin American research is well-placed to provide evidence that is both regionally specific and globally relevant. Developing longitudinal cohorts and integrating prevention into public health systems would help address the region’s current underrepresentation and strengthen its role in shaping international strategies against neurodegeneration, dementia, and related disorders.

In summary, the convergence of recent Latin American evidence [81,82,83,84] with international frameworks [11,12,85] highlights an urgent scientific and public health priority: neurodegeneration must be understood and addressed throughout the lifespan. The reviewed studies show that biomarkers of adversity, including HPA axis dysregulation, chronic inflammation, and epigenetic changes, are directly linked to the molecular mechanisms underlying neurodegenerative disorders in late life.

The next phase of research involves creating long-term pediatric cohorts in underrepresented regions, with a systematic inclusion of psychosocial factors and multi-omics methods. Such efforts are essential for discovering predictive biomarkers, mapping pathways of vulnerability and resilience, and developing culturally and contextually appropriate prevention strategies.

## 8. Limitations

This narrative review has several limitations that should be acknowledged. First, there is a lack of robust longitudinal studies to validate the proposed associations across all ND domains, and most available data come from cross-sectional studies or rely on small or region-specific samples, which limit causal inference and generalizability. Additionally, the interpretation of biomarker findings remains limited by the high heterogeneity of study designs and the presence of unmeasured confounding factors such as nutrition, environmental exposures (e.g., pollution and heavy metals), genetic background, and socioeconomic conditions—factors that influence both adversity and positive childhood experiences, along with their corresponding biological outcomes. Furthermore, although growing evidence supports the association between ACEs and stress-related biological pathways, many identified biomarkers still require multi-omics validation to confirm their reproducibility and translational relevance. Future longitudinal and integrative studies, incorporating clinical, genomic, and environmental data, will be essential to unravel these complex interactions.

## 9. Conclusions

Biomarkers of adversity directly reflect the molecular mechanisms involved in late-life neurodegenerative disorders. However, further research is still needed. Reframing neurodegeneration from a lifespan perspective is not only a conceptual shift but also a scientific imperative. By intervening early in childhood rather than waiting until late adulthood, both Latin America and the global community can effectively reduce the future burden of dementia and related disorders while encouraging healthier brain development across generations.

## Figures and Tables

**Table 1:** Biomarkers, signaling pathways, and mechanistic implications linking ACEs and neurodegeneration.

Mechanistic Process	Signaling Pathway or Molecular Axis/Cellular Effect/Mechanism Relevance in Neurodegeneration	References
Tau phosphorylation	Driven by GC–GR signaling through GSK3β and CDK5, leading to tau hyperphosphorylation, microtubule destabilization, and neurofibrillary tangle formation, contributing to neuronal dysfunction in AD, FTD, and DLB.	[42,43,44,45,46,47,48,49,54]
FKBP5 methylation/expression	GC–GR activation alters FKBP5 and downstream NF-κB/HPA-axis feedback, enhancing stress reactivity and maladaptive inflammatory priming that increases neuroinflammatory vulnerability in AD and PD.	[42,43,45,54]
Aβ accumulation	GC–GR regulation of BACE1 and HPA-axis stress signaling promotes excess amyloid-β production and impaired clearance, facilitating plaque deposition and synaptic loss in AD and DLB.	[43,45,49]
α-synuclein aggregation	ROS-induced NF-κB/TLR2–mediated microglial activation amplifies cytokine signaling and proteostatic failure, accelerating α-syn aggregation and its propagation through vulnerable neural circuits in PD and DLB.	[49,50,56]
Oxidative stress (8-OHdG, MDA)	Activation of NF-κB, MAPK, and mitochondrial ROS pathways induces DNA oxidation, lipid peroxidation, and impaired repair, representing a shared mechanism that accelerates aging processes and pathological spread in AD, PD, MS.	[42,43,45,50,55]

Abbreviations: Aβ: Amyloid-β; AD: Alzheimer’s disease; α-Syn: Alpha-synuclein; BACE1: Beta-secretase 1; CDK5: Cyclin-dependent kinase 5; FKBP5: FK506 binding protein 5; DLB: Dementia with Lewy bodies; FTD: Frontotemporal Dementia; GC: Glucocorticoids; GR: Glucocorticoid receptor; GSK3β: Glycogen synthase kinase-3 beta; HPA: Hypothalamic–pituitary–adrenal (axis); MAPK: Mitogen-activated protein kinase; MDA: Malondialdehyde; NF-κB: Nuclear factor kappa-light-chain-enhancer of activated B cells; PD: Parkinson’s disease; ROS: Reactive oxygen species; TLR2: Toll-like receptor 2.

**Table 2:** Stress-Related Biomarkers related to ACEs and Neurodegeneration.

Biomarker	Sample	Biological Pathway/Mechanism	Representative Neurodegenerative Conditions	Mechanistic Link to Early-Life Adversity	References
Cortisol (HCC, plasma)	Hair, plasma	Dysregulation of the hypothalamic–pituitary–adrenal (HPA) axis, resulting in chronic hypercortisolemia or attenuated stress reactivity	AD, PD, MS	Chronic HPA overactivation following early stress induces hippocampal atrophy, neuronal loss, and tau phosphorylation. Elevated HCC reflects cumulative adversity and predicts cognitive decline.	[25,41,58,59,60,61]
IL-6, TNF-α, CRP	Plasma/PET neuroimaging	Pro-inflammatory cytokines and acute-phase protein reflecting systemic and central neuroinflammation via NF-κB and JAK–STAT activation; associated with microglial priming and increased TSPO-PET signal	AD, PD, MS	Early adversity induces chronic immune activation and glucocorticoid resistance, sustaining elevated IL-6 and TNF-α levels, microglial activation, and neuroinflammatory aging.	[27,37,38,39,40,41]
FKBP5 (mRNA expression)	Blood	Glucocorticoid receptor co-chaperone that modulates HPA feedback and stress sensitivity	AD, PD, FTD	Early stress alters FKBP5 methylation and expression, increasing GR sensitivity and inflammatory signaling, facilitating tau hyperphosphorylation and neuronal vulnerability.	[27,30,42,43,45,54,62]
8-OHdG, MDA, 2-isoprostane, telomere shortening, mtDNA depletion	Plasma, lymphocytes (DNA/RNA via qPCR)	Oxidative stress and mitochondrial damage markers reflecting DNA and lipid oxidation and telomere attrition	AD, PD, MS	Chronic toxic stress accelerates cellular aging, oxidative injury, and mitochondrial dysfunction. Promotes amyloid-β deposits, tau and α-synuclein aggregation, and demyelination.	[42,43,45,50,55]
NfL (Neurofilament light chain)	Serum, plasma	Marker of axonal injury and neurodegeneration	AD, PD, MS, ALS	Elevated NfL levels correlate with ACE exposure and predict structural brain changes and cognitive decline before symptom onset.	[1,52,57,63,64,65]
GFAP (Glial fibrillary acidic protein)	Serum, plasma	Marker of astrocytic reactivity and chronic neuroinflammation	AD, MS, DLB	Persistent stress promotes astroglial activation; elevated GFAP indicates neuroinflammation and glial injury associated with early adversity and dementia risk.	[1,63,65]
Protein aggregation markers (p-tau181/217, Aβ42/40, α-synuclein)	CSF, plasma	Proteostatic imbalance, ER stress, and impaired autophagy–proteasome activity	AD, PD, DLB	Stress-induced mitochondrial dysfunction and impaired proteostasis lead to accumulation of misfolded proteins; stress accelerates tau propagation and α-synuclein spread.	[43,45,49,50,56,66,67]
Neuroimaging biomarkers (TSPO-PET, MRI)	PET, MRI	Visualization of neuroinflammation and stress-related cortical and hippocampal alterations	AD, PD, MS	Early adversity increases TSPO-PET signal (microglial activation) and reduces cortical and hippocampal volumes, reflecting neuroinflammatory remodeling and stress-related atrophy.	[1,38,39]

## Data Availability

No new data were created or analyzed in this study. Data sharing is not applicable to this article.

## Abbreviations

The following abbreviations are used in this manuscript:

ACEs	Adverse Childhood Experiences
Aβ	Beta-amyloid peptide
ALS	Amyotrophic Lateral Sclerosis
AOPP	Advanced Oxidation Protein Products
BBB	Blood–Brain Barrier
BCEs	Benevolent Childhood Experiences
BDNF	Brain-Derived Neurotrophic Factor
CRP	C-reactive Protein
CSF	Cerebrospinal Fluid
DLB	Dementia with Lewy Bodies
DNA	Deoxyribonucleic Acid
EDSS	Expanded Disability Status Scale
EBV	Epstein–Barr Virus
EAI	Early Adverse Experiences (for Spanish references, optional)
FTD	Frontotemporal Dementia
GFAP	Glial Fibrillary Acidic Protein
GR	Glucocorticoid Receptor
HPA	Hypothalamic–Pituitary–Adrenal Axis
HD	Huntington Disease
IL	Interleukin
IL-6	Interleukin-6
IL-10	Interleukin-10
IJMS	International Journal of Molecular Sciences
LBD	Lewy Body Dementia (alternative term for DLB)
MS	Multiple Sclerosis
mtDNA	Mitochondrial DNA
NDs	Neurodegenerative Diseases
NF-κB	Nuclear Factor Kappa-light-chain-enhancer of Activated B Cells
NfL	Neurofilament Light Chain
NRF2	Nuclear Factor Erythroid 2-Related Factor 2
OPA1	Optic Atrophy Protein 1
PCEs	Positive Childhood Experiences (if used synonymously with BCEs)
PD	Parkinson’s Disease
PET	Positron Emission Tomography
PTSD	Post-Traumatic Stress Disorder
ROS	Reactive Oxygen Species
SOD1	Superoxide Dismutase 1
suPAR	Soluble Urokinase Plasminogen Activator Receptor
Tau181	Phosphorylated Tau at Threonine 181
Tau217	Phosphorylated Tau at Threonine 217
TNF-α	Tumor Necrosis Factor Alpha
TRAP	Total Radical-Trapping Antioxidant Parameter
TSPO	Translocator Protein
VDAC1	Voltage-Dependent Anion Channel 1
